# Hostile and threatening interpretation biases in adolescent inpatients are specific to callous-unemotional traits and social anxiety

**DOI:** 10.1007/s00787-023-02227-3

**Published:** 2023-05-31

**Authors:** Anna L. Dapprich, Laura M. Derks, Martin Holtmann, Wolf-Gero Lange, Tanja Legenbauer, Eni S. Becker

**Affiliations:** 1 Behavioural Science Institute, Radboud University, Nijmegen, The Netherlands; 2 LWL-University Hospital Hamm for Child and Adolescent Psychiatry, Psychotherapy and Psychosomatics, Ruhr-University Bochum, Bochum, Germany

**Keywords:** Cognitive distortions, Adolescence, Social information processing, Internalizing disorders, Externalizing disorders

## Abstract

Psychopathological syndromes, such as disruptive behavior and anxiety disorders in adolescence, are characterized by distorted cognitions and problematic behavior. Biased interpretations of ambiguous social situations can elicit both aggressive and avoidance behavior. Yet, it is not well understood whether different interpretation biases are specific to different syndromes, or whether they can co-occur. We assessed both hostile and threatening interpretation biases in identical social situations, and proposed that they are uniquely related to callous-unemotional (CU) traits and social anxiety, respectively. We also explored the role of gender and age herein. The sample consisted of 390 inpatients between 10 and 18 years of age with a variety of psychiatric disorders. Hostile and threatening interpretations were assessed with the Ambiguous Social Scenario Task (ASST) consisting of 10 written vignettes. Both CU-traits and social anxiety were assessed with self-report questionnaires. Results showed that, overall, CU-traits were related to more hostile interpretations, whereas social anxiety was related to more threatening interpretations. In addition, in boys, hostile and threatening interpretations correlated significantly positive with each other. Age was not related to interpretation biases. Together, these results generally support the content-specificity of interpretation biases in concepts relevant to disruptive behavior disorders and anxiety disorders, and indicate that different interpretation biases can co-occur specifically in boys.

**Supplementary Information:**

The online version contains supplementary material available at 10.1007/s00787-023-02227-3.

## Introduction

A wealth of theories and research have shown that distorted interpretations of social situations have a causal and maintaining role in both disruptive behavior disorders and anxiety disorders [1–5]. The core symptom of disruptive behavior disorders, such as oppositional defiant and conduct disorder, is aggressive behavior, which has been associated with the tendency to interpret ambiguous social situations as hostile, i.e., *hostile interpretation bias* [6–9]. Anxiety disorders have been associated with the tendency to interpret ambiguous situations as threatening, i.e., *threatening interpretation bias* [10, 11]. However, some studies found that aggressive children also showed threatening interpretation bias, and that anxious children also showed hostile interpretation bias [11–13]. Albeit counterintuitive, aggression and anxiety are closely related to each other; they are both part of the interwoven fight-or-flight system and they can reinforce each other [14–17]. Yet, hostile and threatening interpretation biases are usually assessed with separate, unrelated tasks. Research that studied different interpretation biases together in the same situation, and in relation to both disruptive behavior and anxiety disorders is scarce. Therefore, it is unclear whether interpretation biases are specific to distinct syndromes, or whether they can co-occur [13, 18–21]. In the current study, we assessed both hostile and threatening interpretation biases of identical social situations, and examined whether they are uniquely linked to callous-unemotional (CU) traits and social anxiety, respectively, in inpatients of children and adolescents with a variety of psychiatric disorders.

CU-traits and social anxiety are clinically and developmentally relevant concepts in disruptive behavior and anxiety disorders, respectively. CU-traits are characterized by shallow affect, disregard for others and indifference towards performance in school and relationships. Children and adolescents with both elevated levels of CU-traits and disruptive behavior disorders are at highest risk for severe behavioral problems, treatment resistance, as well as antisocial personality disorder and psychopathy in adulthood [22–24]. Social anxiety is characterized by elevated fear of social evaluation [25]. It usually develops in early adolescence and, if left untreated, increases the risk on chronic anxiety and mood disorders later in life [26, 27]. On the behavioral level, CU-traits are strongly related to aggression and social anxiety is strongly related to avoidance. However, social anxiety has also been related to reactive aggression described as impulsive reactions to perceived threat [15]. CU-traits, in turn, are rather related to instrumental aggression described as deliberate acts to reach a certain goal [1, 28, 29]. CU-traits have also been related to avoidance, such as truancy [30, 31]. Thus, the behavioral outcomes of social anxiety and CU-traits can be very similar, but the underlying processes leading to aggression or avoidance are likely to differ. For instance, CU-traits are associated with hypoactive physiological responding to threat [32], while social anxiety is associated with hyperactive physiological responding to threat [33]. The interpretation of threat could provide more information about the cognitive processes contributing to aggression and avoidance in CU-traits and social anxiety.

Previous research on the relationship between CU-traits and interpretation biases is inconclusive. A common way to investigate interpretation biases in children and adolescents are written vignettes describing ambiguous situations [34]. In a clinical sample of children with disruptive behavior disorders, no link between CU-traits and hostile interpretation bias of ambiguous situations was found [35]. In college students, CU-traits were related to stronger hostile interpretations [36]. Surprisingly, CU-traits have also been related to more threatening interpretation bias of social situations in delinquent adolescents [37]. Thus, there is some support for a link between CU-traits and interpretation biases. An investigation of different interpretation biases together might provide more clarity.

Interpretation biases in childhood anxiety have been well-examined. According to the *content-specificity hypothesis* [38], the strength of interpretation biases increases when the described scenario matches a particular fear [20, 21]. For example, in a clinical sample of children with different anxiety disorders, children with higher levels of separation anxiety rated separation scenarios as the most threatening, whereas children with higher levels of social anxiety rated ambiguous social scenarios as the most threatening [39]. Using written vignettes, it has been well-established that these threatening interpretation biases of social situations increase, as the severity of social anxiety increases in both clinical and community samples [40–42], for conflicting results see [43]. Yet, it is unclear whether the content-specificity hypothesis holds true when comparing different interpretation biases of the same situations across concepts relevant to disruptive behavior and anxiety disorders.

The current study investigated whether hostile and threatening interpretation biases of the same social situations are uniquely related to CU-traits and social anxiety, respectively, in inpatients of children and adolescents with different psychiatric disorders. By better understanding the cognitive processes accompanying disruptive behavior and anxiety in childhood, current prevention and intervention paradigms of childhood psychopathology could be improved. Based on some previous findings [36] and the content-specificity hypothesis [38], we expected that CU-traits and social anxiety are distinctly related to hostile and threatening interpretation biases, respectively. Hostile and threatening interpretation biases were assessed with the newly developed Ambiguous Social Scenario Task (ASST), which measures both interpretation biases simultaneously in response to written vignettes of ambiguous social scenarios. This paradigm has previously been validated in college students [36]. Given that CU-traits decrease with age and are more prevalent in boys [44], whereas social anxiety increases with age and is more prevalent in girls [21, 41, 43], we controlled for age and gender differences when analyzing the link between CU-traits, social anxiety and biases.

## Methods

### Participants

Four-hundred-and-one inpatients of the LWL-University Hospital Hamm in Germany participated. Patients who were older than 18 years of age (*n* = 3) or had a diagnosis of the schizophrenia spectrum and other psychotic disorders (*n* = 8) were excluded. The final sample consisted of 390 inpatients (248 girls) between 10 and 18 years of age (M = 14.6 years, SD = 1.9). The most common primary diagnosis was Major Depressive Disorder (*n* = 174). The majority of the sample was diagnosed with comorbid disorders (*n* = 248). See Table 1 for the sample characteristics.

**Table 1:** Sample characteristics

Variable	Final sample (*N* = 390)
Age *M (SD)*	14.6 (1.9)
Female *n (%)*	248 (64)
No. of diagnoses M (SD)	2.24 (0.1)
Primary diagnosis *n (%)*	
Major Depressive Disorder (F32.-, F33.-)	174 (44.3)
Mixed Disorder of Conduct and Emotion (F92.-)	93 (23.7)
Substance Abuse Disorder (F10.2, F11.2, F12.2, F15.2)	52 (13.2)
Comorbidities *n (%)*	248 (63.1)
No. of comorbidities *M (SD)*	1.24 (0.1)

Diagnoses are based on the International Classification of Disease 10th revision (ICD-10)

## Measurements

### Ambiguous social scenario task—youth version (ASST—youth version; adapted from [36])

The ASST—youth version measures hostile, threatening and neutral interpretations in social situations. It contains 10 social scenarios, each with a hostile, a threatening and a neutral interpretation. Participants have to indicate for each situation how likely each of the three interpretations would come to their mind on a visual analogue scale from 0% (“very unlikely”) to 100% (“very likely”). Thus, all interpretations could be rated as very likely for the same scenario. An example situation is “You are asked something by the teacher in class. The teacher interrupts you in the middle of your answer”. The answer options state (1) “Probably she found my answer boring.” (threatening interpretation), (2) “Stupid teacher!” (hostile interpretation) and (3) “Apparently, I told her everything she wanted to hear.” (Neutral interpretation). The whole task can be found in the supplementary material (SI 1).

For the analyses, separate mean scores for threatening, hostile and neutral interpretations were used. A validation study in students showed good convergent and discriminant validity for both threatening and hostile interpretations [36]. In the current study, the internal consistencies ranged from acceptable for the neutral (*α* = 0.69) and hostile interpretations (*α* = 0.77) to good for the threatening subscale (*α* = 0.84).

### Spence childrens anxiety scale (SCAS-D; [45])

The SCAS-D measures self-reported levels of anxiety based on six different subscales, i.e., social phobia, panic disorder, agoraphobia, generalized anxiety disorder, obsessive–compulsive disorder, separation anxiety disorder, and specific phobias. It consists of 38 items, which are rated on a 4-point Likert scale ranging from 0 (“never”) to 3 (“always”). Previous research supported the 6-factor structure of the SCAS-D in terms of excellent validity and internal consistency [46]. For the present study, only the social anxiety scale was used consisting of 6 items (e.g., “I worry what other people think of me”). Its internal consistency was high in our sample (*α* = 0.86).

### Inventory of callous-unemotional traits (ICU; [47])

The ICU measures callous-unemotional (CU) traits. It consists of 24 items and is rated on a 4-point Likert scale ranging from 0 (“not at all true”) to 3 (“definitely true”). Three factors of good psychometric properties can be distinguished, which are called Callousness, Uncaring and Unemotional [48, 49]. Example items are “I do not care if I get into trouble” and “I do not show my emotions to others”. For the current study, the total sore was used, which had an acceptable internal consistency (*α* = 0.76).

#### Procedure

Patients who were admitted to the clinic within a pre-defined period of 6 months filled in a range of questionnaires as part of the diagnostic intake routine. Here, only the questionnaires that are relevant for the current purposes are described. The use of the data for the current study was approved by the local medical-ethical committee (No.: 4359–12). The whole procedure took about 1 h. Participation was not rewarded.

#### Statistical approach

The main research question, whether self-reported social anxiety and CU-traits were uniquely related to threatening and hostile interpretations, respectively, was examined with Pearson correlation coefficients, as well as multivariate multiple regression and univariate multiple regression analyses (complete cases only, *n* = 386). Multivariate regression controls for inflated Type I error of several univariate regressions, and takes into account that outcomes and predictors are interrelated. Univariate regressions are necessary to interpret the results for each outcome separately. Significant effects were followed-up with Welch’s robust two sample t-tests, plots and simple slope analyses.

Data were prepared and analyzed using R (version 4.0.3; [50]) and RStudio (version 2022.07.1; [51]). The correlation coefficients were computed using the function *corr.test* of the package *psych* (version 2.2.9; [52]). Holm adjustment was used to control for multiple testing. Multivariate multiple hierarchical regression analyses with both hostile and threatening interpretations as outcome were conducted using the function *lm* of the *stats* package (version 4.0.3; [50]). In the first step, age and gender were entered as predictors (i.e., basic model). In the second step, main effects for both CU-traits and social anxiety, as well as their respective interactions with both age and gender were added (i.e., full model). The continuous predictors were standardized. The function *anova* of the *psych* package was used to compare the fit of the basic and full model (version 2.2.9; [52]). The functions *Manova* and *summary* were used to interpret the results of both multivariate and univariate regression analyses. Follow-up t-tests were computed using the function *t.test* of the *stats* package (version 4.0.3; [50]). Significant interaction effects were plotted with the function *ggplot* of the package *ggplot* (version 3.3.6; [53]) and simple slopes were computed using the function *sim_slopes* of the package *interactions* (version 1.1. 5; [54]).

#### Transparency statement

The current project has been pre-registered on osf.io (https://osf.io/7zxyf and https://osf.io/y532q?view_only=8f97db4aff3e4381aa9cda79423cd9cb). Note that deviations from the first pre-registration are specified in the second pre-registration. Most importantly, we originally planned to not only study self-reported CU-traits and social anxiety, but also diagnoses of disruptive behavior disorders and social anxiety disorder. However, the number of patients that received social anxiety disorder as a main diagnosis was small (*n* = 7). Since analyses with this group size wouldn’t yield meaningful results, we focused on self-reported CU-traits and social anxiety only.

## Results

### Relationships between CU-traits, social anxiety, and interpretation biases

Children with higher levels of CU-traits were more likely to interpret ambiguous situations as hostile, whereas children with higher levels of social anxiety were more likely to interpret the very same situations as threatening. CU-traits correlated significantly positive with hostile interpretations (*r* = 0.38, *p* < 0.001), and social anxiety correlated significantly positive with threatening interpretations (*r* = 0.68, *p* < 0.001)*.* Furthermore, CU-traits correlated significantly negative with both threatening interpretations (*r* = − 0.14, *p* = 0.04) and social anxiety (*r* = − 0.19, *p* = 0.001).

### Multivariate multiple hierarchical regression analysis for both interpretation biases

The full model including age, gender, CU-traits and social anxiety improved the model fit of the basic model including only age and gender significantly, Pillai’s trace (*V*) = 0.52, *F*(16, 750) = 16.34, *p* < 0.001.^1^ Significant main effects for gender *V* = 0.04, *F*(2, 374) = 7.71, *p* < 0.001, CU-traits, *V* = 0.12, *F*(2, 374) = 24.32, *p* < 0.001, and social anxiety, *V* = 0.32, *F*(2, 374) = 86.95, *p* < 0.001, were found. Furthermore, an interaction between gender and social anxiety was found, *V* = 0.02, *F*(2, 374) = 3.34, *p* = 0.02. Next, univariate regression analyses with only hostile and threatening interpretations as outcomes were inspected to interpret the effects.

### Univariate regression analysis for threatening interpretation bias only

The model for threatening interpretations was significant *F*(11, 375) = 32.74, *p* < 0.001 and the predictors explained 47.49% of the variance, 95% bootstrapped Confidence Interval (CI) [41.80, 54.29]. A significant main effect for social anxiety on threatening interpretations was found (*β* = 0.62, *p* < 0.001) indicating that threatening interpretations increased as a function of self-reported social anxiety for both genders. Furthermore, a significant main effect of gender on threatening interpretations was found (*β* = − 0.13, *p* = 0.007). *T*-tests indicated that threatening interpretations were significantly higher in girls than in boys, *t*(310.18) = − 9.17, *p* < 0.001, *Cohen’s d* = − 0.96. See Table 2 for the descriptive statistics per gender.

**Table 2:** Descriptive statistics and gender differences of all measurements for the final sample (*N* = 390)

	*n*	Boys		Girls			*p*	*Cohen’s d*
		M (SD)	Range	*n*	M (SD)	Range		
Callous-unemotional traits	142	28 (9)	9–49	247	24 (8)	8–51	< 0.001	0.50
Social anxiety	141	6 (4)	0–18	246	11 (5)	0–18	< 0.001	1.18
Hostile interpretation bias	142	35 (16)	2–75	248	29 (17)	0–90	< 0.001	0.38
Threatening interpretation bias	142	39 (19)	0–86	248	58 (20)	3–99	< 0.001	0.96
Neutral interpretations	142	40 (16)	1–82	248	33 (14)	2–72	< 0.001	0.47

Gender differences were tested with Welch’s *t*-test

### Univariate regression analysis for hostile interpretation bias only

The model for hostile interpretations was significant, *F*(11, 375) = 6.62, *p* < 0.001 and the predictors explained 13.8% of the variance, 95% bootstrapped CI [9.82, 22.34]. A significant main effect for CU-traits on hostile interpretations was found (*β* = 0.35, *p* < 0.001) indicating that hostile interpretations increased significantly as a function of CU-traits. Furthermore, a significant main effect for gender on hostile interpretations was found (*β* = 0.15, *p* = 0.02). *T*-tests indicated that hostile interpretations were significantly higher in boys than in girls, *t*(306.62) = 3.66, *p* < 0.001, *Cohen’s d* = . 38. See Table 2.

**Fig. 1:**
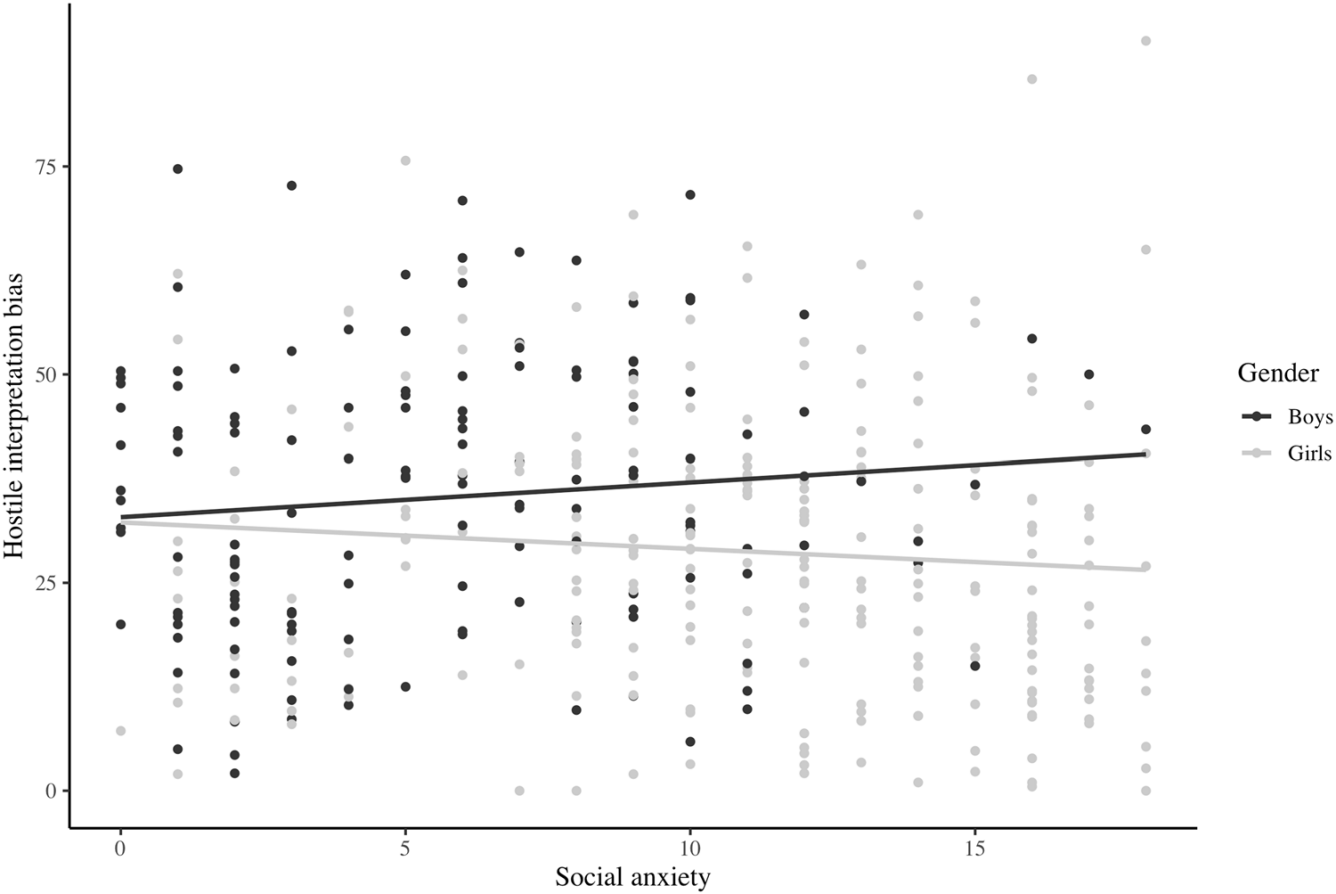
Significant interaction between social anxiety and gender on hostile interpretation bias (*n* = 386). Note*.* Follow-up analyses showed that the increase and decrease in hostile interpretation bias as a function of social anxiety was not significant for boys and girls, respectively

A significant interaction for social anxiety and gender on hostile interpretations was found (*β* = 0.13, *p* = 0.04). A plot showed that with increasing levels of social anxiety, hostile interpretations were more likely in boys, but less likely in girls (see Fig. 1). However, neither simple slopes nor correlations for boys and girls separately were significant. This indicates that differences in social anxiety between boys and girls were, after all, not related to hostile interpretations (*p*’s > 0.05).

### Exploration of gender differences

The significant effects of gender on both hostile and threatening interpretations led us to further explore its role for the variables of interest. As shown in Table 2, all measurements differed significantly for girls and boys. Furthermore, Pearson’s correlations for girls and boys separately showed that the correlations between CU-traits and hostile interpretations (*r* = 0.34 for girls and* r* = 0.36 for boys), as well as between social anxiety and threatening interpretations (r = 0.66 for girls and *r* = 0.50 for boys) were still significant and positive (all *p*’s < 0.001). The correlations between self-reported CU-traits and social anxiety were not significant for girls and boys separately (*p*’s > 0.05). Interestingly, threatening and hostile interpretations correlated significantly positive in boys (*r* = 0.36, *p* < 0.001), but not in girls (*r* = − 0.01, *p* > 0.05). See Table 3 for the correlation matrix per gender.

**Table 3:** Correlation matrix between all measurements per gender

	1	2	3	4	5
1. Callous-unemotional traits	–	− 0.09	0.34***	− 0.08	– 0.20*
2. Social anxiety	− 0.11	–	− 0.08	0.66***	− 0.21**
3. Hostile interpretation bias	0.36***	0.11	–	− 0.01	0.21**
4. Threatening interpretation bias	0.01	0.50***	0.36***	–	− 0.14
5. Neutral interpretations	− 0.06	0.06	0.10	0.15	–

Values above diagonal depict correlations for girls, values below the diagonal depict values for boys

***p < 0.001

**p < 0.01

*p < 0.05

## Discussion

The current study investigated whether hostile and threatening interpretation biases are specific to callous-unemotional (CU) traits and social anxiety, respectively, in adolescent inpatients with a variety of different psychiatric disorders. Our results suggest that CU-traits are uniquely related to hostile interpretation bias, and that social anxiety is uniquely related to threatening interpretation bias. Although, boys who showed more hostile interpretation bias, also used more threatening interpretation bias.

In line with the content-specificity hypotheses, CU-traits and social anxiety were related to distinct interpretation biases of the same situations. Adolescent inpatients who reported more callousness were more likely to interpret social situations as hostile, whereas adolescent inpatients who reported more fear of social evaluation were more likely to interpret the same situations as threatening. This is particularly important as research has shown that different interpretations of social cues might underlie different behavioral outcomes, such as aggression and avoidance [1–3, 5]. Thus, CU-traits and social anxiety might be characterized by a distinct cognitive processing of threat, which might steer different behavioral problems.

The manifestation of interpretation biases differed across boys and girls. First, both CU-traits and hostile interpretation bias were higher in boys, and social anxiety and threatening interpretation bias were higher in girls. This is in line with the common finding that boys show more disruptive behavior [8, 44, 55], and that girls more often have (social) anxiety disorders [41, 56]. Second, boys showed more hostile interpretation bias with increasing levels of social anxiety, while girls showed less. This is in line with research showing that the link between anxiety and aggression is more pronounced in boys [17]. However, this effect diminished at gender-specific post-hoc analyses. Finally, boys who showed more hostile interpretation bias, also showed more threatening interpretation bias, while girls did not. Previous research has also found hostile and threatening thoughts to co-occur, but did not examine gender differences [57, 58]. This suggests that hostile and threatening interpretation biases can co-occur in boys independent of CU-traits and social anxiety. Ambiguous events might activate a general negative schema [59]. Stronger biases might then be a sign of a higher general psychopathological symptom level.

In contrast to most previous research, we did not find an effect of age on interpretation biases [21, 44]. A possible explanation for the absence of a linear effect might be that interpretation biases follow a curvilinear course across age, as do both social anxiety and CU-traits. To be precise, a peak in social anxiety and CU-traits has been suggested as a normative part of puberty [49, 60, 61]. Indeed, the age range of our sample (10–18 years of age) covered different developmental stages from childhood to late adolescence. Longitudinal research is needed to determine the developmental course of interpretation biases across age.

The current study comes with several strengths and limitations. A strength is the large clinical sample, which allowed us to investigate clinically relevant concepts across diagnostic categories in a well-powered study. Furthermore, we replicated and extended a previous validation study of the Ambiguous Social Scenario Task (ASST) in college students [36]. That is, we again found good psychometric properties of the ASST, as well as the expected relationships between hostile and threatening interpretation biases and clinically relevant concepts for disruptive behavior and anxiety. However, due to too little group sizes, we could not examine the roles of psychiatric disorders in interpretation biases. The inclusion of diagnostic categories might have increased the relatively low explained variance of the model on hostile interpretation biases in the current study. By examining different interpretation biases in relation to different psychiatric disorders, we would better understand whether interpretation biases are disorder-specific or transdiagnostic mechanisms [18]. Furthermore, future research should study the link between interpretation biases and actual social behavior to gain a more complete picture of the steps involved in social information processing and its consequences for behavior.

The current study represents a first step examining different interpretation biases together in relation to psychiatric concepts relevant to disruptive behavior disorders and anxiety disorders in childhood and adolescence. A combined investigation of distinct interpretation biases in a clinical sample with a multitude of diagnoses is crucial to disentangle underlying mechanisms of distinct problematic behavior, as well as to identify transdiagnostic characteristics of psychological disorders. The current results suggest that interpretation biases can be differentiated in terms of callous versus anxious cognitive processing of social threat, and that gender might be an important factor to take into account. On the long term, this knowledge might help to develop (gender) tailored interventions focusing on the modification of interpretation bias to improve treatment outcomes for callous and anxious youth.

### Supplementary Information

Below is the link to the electronic supplementary material.

Supplementary file1 (DOCX 48 KB)

## Data Availability

The anonymized data can be requested from the first author upon reasonable request. Part of the material is included in the supplementary material.

## Footnotes

1 As an exploratory analysis, we also tested a third model that included quadratic main effects for both CU-traits and social anxiety. However, our findings indicate that this model did not significantly improve the model fit of the previous model, *V* = .05, *F*(16, 734) = 16.34, *p* = .297.

## Code availability

The used software is referenced in text.
