# Supplementary material for: Hostile and threatening interpretation biases in adolescent inpatients are specific to callous-unemotional traits and social anxiety
Source: Eur Child Adolesc Psychiatry. 2023 May 31;33(4):1143–50. doi: 10.1007/s00787-023-02227-3 (PMC11032296; doi:10.1007/s00787-023-02227-3)
Supplement: Supplementary file 1 — Supplementary file1 (DOCX 48 KB) [file 787_2023_2227_MOESM1_ESM.docx]

**Supplementary information (SI)**

Title:

Hostile and threatening interpretation biases in adolescent inpatients are specific to callous-unemotional traits and social anxiety

Anna L. Dapprich^1, *
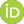
^ https://orcid.org/0000-0002-2208-698X

Laura M. Derks^1,2^
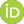
 https://orcid.org/0000-0001-5333-0618

Martin Holtmann^2^
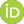
 : 0000-0002-1845-7688

Wolf-Gero Lange^1^
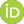
 0000-0002-4236-0819

Tanja Legenbauer^2^
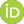
 0000-0002-1580-9991

Eni S. Becker^1^
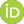
 https://orcid.org/0000-0003-3524-426X

^1^Behavioural Science Institute, Radboud University, The Netherlands

^2^LWL-University Hospital Hamm for Child and Adolescent Psychiatry, Psychotherapy and Psychosomatics, Ruhr-University Bochum, Germany

*Corresponding author

Radboud University

Behavioural Science Institute

6500 HB Nijmegen

The Netherlands

Email: anna.dapprich@gmail.com

**SI 1: Ambiguous Social Scenario Task – youth version**

*Instructions*

In this task, different situations are described and three possible thoughts are presented. Indicate for each thought how likely it would come to your mind in that respective situation.

There is no right or wrong in this task, trust your feeling and answer spontaneously.

*Situation 1)*

You hang out with a group. When you start talking, nobody looks at you

Would you think …

a. They did not expect me to start talking.

b. I poorly timed the moment to say something.

c. They just don’t look at me by chance but they are certainly interested in what I say.

*Situation 2)*

You are having a doctor’s appointment. The doctor’s assistant asks you to sit down and wait. While you’re waiting, you hear the assistant saying that there is an annoying child in the waiting room.

Would you think …

a. Indeed, I should have been more friendly.

b. I find the woman annoying, too.

c. Doctor’s assistants are just grumpy.

*Situation 3)*

You are asked something by the teacher in class. The teacher interrupts you in the middle of your answer.

Would you think …

a. Probably she found my answer boring.

b. Stupid teacher.

c. Apparently, I told her everything she wanted to hear.

*Situation 4)*

You bump into a classmate in the street. As he sees you, he laughs.

Would you think …

a. What an overly nice person.

b. He greets everyone like this.

c. Does he laugh at me?

*Situation 5)*

Some people you know are looking at you and start talking to each other.

Would you think …

a. They are looking into my direction randomly.

b. They are probably gossiping about me.

c. They look really stupid.

*Situation 6)*

You post a new profile picture on Facebook. When you check in the evening, nobody has liked your photo.

Would you think …

a. That's embarrassing! I will just delete my picture quickly.

b. Probably no one had time to like my picture, yet.

c. I won't like pictures of others anymore either.

*Situation 7)*

You asked a question in a WhatsApp group chat. After a while, you see that everybody has read your message, but no one has responded.

Would you think …

a. They were busy and didn't have time to answer.

b. They find my question stupid and annoying.

c. Those people are just lazy, I won't reply to their questions anymore either.

*Situation 8)*

You and your friend had agreed upon sleeping in the same room during the next school trip. Lately she/ he said to another classmate that they would share a room during that school trip.

Would you think …

a. I am probably too boring.

b. I won’t invite either of them to my birthday party.

c. Maybe all three of us can share a room together.

*Situation 9)*

During school break you see a classmate sitting alone. You head towards him and ask if you can sit next to him. He just says “No”.

Would you think …

a. Alright, stay there alone. I have enough other friends here.

b. He is probably waiting for someone or he's having a bad day. At least I've tried.

c. Why doesn’t he like me? I hope nobody saw that I just got the push.

*Situation 10)*

You tell something very personal to a friend and stress that she shouldn’t tell anyone. A few weeks later you realize that more of your friends know about it. You talk to your friend and she says: “Oh, I don’t know. We just talked about it and I didn’t think it was a big deal”.

Would you think …

a. Now that all of them know, they are definitely going to gossip about me.

b. I would have preferred if it had stayed between us but we're all friends, so it'll be fine.

c. I will also tell her secrets
